# The Hispanic Paradox: A Moderated Mediation Analysis of Health Conditions, Self-Rated Health, and Mental Health among Mexicans and Mexican Americans

**DOI:** 10.1080/21642850.2022.2032714

**Published:** 2022-02-09

**Authors:** Cindy M. Hernandez, Oswaldo Moreno, Isis Garcia-Rodriguez, Lisa Fuentes, Tamara Nelson

**Affiliations:** a Virginia Commonwealth University, Richmond, VA, USA; b Rutgers University - Camden, Camden, NJ, USA

**Keywords:** Hispanic Paradox, Immigrant Health Paradox, mental health, Mexican, immigrants

## Abstract

This study investigates how mediating (e.g. history of health conditions) and moderating (e.g. self-rated health) factors are associated with nativity status on depression and anxiety in Mexican immigrants. Using data from the 2019 National Health Interview Survey (NHIS), results found a significant direct association between nativity status and anxiety and depression. Additionally, the association between nativity status and mental health was mediated by the history of health conditions, and self-rated health was a significant moderator in both mediation models. Study findings are discussed within the context of barriers to care, current literature, and strengths-based interventions. Future research can expand upon these findings by examining the specific types of physical and mental health conditions that may support the Hispanic Paradox, as well as how self-efficacy and internal locus of control are associated with the paradox within this population.

Over 60.5 million U.S. residents identify as being of Latinx/Hispanic origin (U.S. Census Bureau, 2021). Although a general sample of Latinx^1^ adults represents a mosaic of ethnic/regional subgroups (e.g. Mexicans, Dominicans, Puerto Ricans, etc.) with important intergroup differences, broad comparisons between Latinx and non-Latinx whites have revealed pressing need-utilization mismatches in mental health and mental healthcare disparities (Olawa, Adebayo, Mokuolu, Umeh, & Omolayo, 2020). The Hispanic Paradox, also referred as the Immigrant Health Paradox, refers to a psychological phenomenon in which immigrants report lower physical and mental health concerns than native born Americans (Casanova & Aguila, 2020; Jimenez, Alegría, Chen, Chan, & Laderman, 2010; Markides & Rote, 2015) in spite of the stressors faced in the U.S. (e.g. language and financial barriers; Jimenez et al., 2010. This has been shown to have a clear association among Latinxs of Mexican origins Cooper et al., 2020. However, research suggests that overtime, mental health outcomes decline and become similar to the general U.S. population Jerant, Arellanes, & Franks, 2008.

Epidemiological surveys and other large-scale studies have demonstrated that Latinx adults show no differences in prevalence rates of mental health disorders when compared to non-Latinx whites Saklofske, Austin, Galloway, & Davidson, 2007; Alegría, Álvarez, & DiMarzio, 2017. However, studies point to lower mental health service utilization rates by Latinx adults in comparison to non-Latinx whites, even after controlling for income and insurance coverage Cooper et al., 2020. Furthermore, studies considering demographic, socioeconomic, and acculturation covariates have found that protective or risk factors greatly vary between Latinx subgroups, pointing toward the need to move toward within-group analyses of psychological distress among Latinxs Olawa et al., 2020; Varin, Baker, Palladino, & Lary, 2019. For example, Barragán, Yamada, Gilreath, and Lizano (2020) found that speaking Spanish, used as a proxy measure for acculturation, buffered psychological distress among Mexican Americans but was associated with an increased risk of psychological distress in Mexican, Central/South American, Puerto Rican, Cuban/Cuban American, and other Latinx participants.

There is a further mental health disparity between Latinx-born immigrants and U.S.-born Latinx Americans (Olawa et al., 2020). Researchers have found that Mexican-born immigrants endorse lower lifetime prevalence rates of depressive disorder, anxiety disorder, and substance use disorders when compared to their U.S.-born Latinx American and acculturated counterparts (Teruya & Bazargan-Hejazi, 2013; Rutter & Brown, 2017; Bunda & Busseri, 2019). Latinx-born and undocumented Latinxs are also less likely than U.S.-born Latinxs to obtain mental health services, alluding to the presence of a complex barrier that influences mental health service use within this population (Teruya & Bazargan-Hejazi, 2013; Bunda & Busseri, 2019; Kimbro, Bzostek, Goldman, & Rodríguez, 2008). Furthermore, a systematic review and meta-analysis sampling migrants across 20 different countries found that immigrants with a shorter duration of stay in their county of migration report greater depression prevalence (Alegría et al., 2008). Another multi-ethnic study found that immigrants and their children show higher risks of depression in comparison to the host population (Moreno & Cardemil, 2018). Overall, literature on the intersection of immigration and mental health outcomes has indicated that immigrants’ risk of depression may decline generationally as they integrate into their host country (Alegría et al., 2008; Moreno & Cardemil, 2018).

In conjunction with previously mentioned research on Mexican immigrants, these findings suggest the presence of unique factors protecting Mexican-origin individuals from negative mental health outcomes. Researchers attempting to decipher the origins of this disparity have emphasized the influence that various moderators (e.g. self-reported health, acculturation levels, and cultural stress; Olawa et al., 2020; Ai, Carretta, & Aisenberg, 2017; National Center for Health Statistics. National Health Interview Survey, 2019) and mediators (e.g. history of health conditions; Varin et al., 2019; Teruya & Bazargan-Hejazi, 2013; Adames & Chavez-Dueñas, 2016) have on the complex relationship between nativity status and mental health in Latinx adults. The purpose of this study was to investigate how mediation (e.g. history of health conditions) and moderation (e.g. self-rated health) factors are associated with nativity status on depression and anxiety in Mexican-origin individuals.

## The Hispanic Paradox & the Biosocial Theory

The overall Hispanic Paradox posits that, when compared to their US-born counterparts and those who remain in their country-of-origin, foreign-born immigrants exhibit greater health outcomes including self-rated health, mortality, reproductive health, BMI, and mental health (Adames, Chavez-Dueñas, Fuentes, Salas, & Perez-Chavez, 2014; Garcini et al., 2020). Several explanations for this health-protective effect abound. A frequently cited form of protection observed within foreign-born immigrant communities is that of socio-cultural protection, which refers to reliance on tightly-packed social networks and immigrant-dense neighborhoods for support (Jones et al., 2018; Reyes, Constantino, Cross, Tan, & Bombard, 2020). Some researchers have concluded that exposure to U.S. society may be deleterious for some immigrant health outcomes due to the stresses of everyday life and acquisition of unhealthy habits that increase with duration of stay in the host country and acculturative processes (Jones et al., 2018; Reyes et al., 2020; Escovar et al., 2018). Despite the abundance of literature exploring the breadth of the Hispanic Paradox, a majority of the work supporting the paradox has revolved around physical health and not mental health outcomes (Garcini et al., 2020).

Although the Salmon Bias Hypothesis (Garcini et al., 2020; Okeke-Ihejirika, Creese, Frishkopf, & Wane, 2020; John, de Castro, Martin, Duran, & Takeuchi, 2012) and the Selection Theories (Adames et al., 2014; John et al., 2012) have been used to understand the Hispanic Paradox, Biosocial Theory (Löwe et al., 2008; Harris & McDade, 2018) may provide a unique perspective in providing a different perspective towards the Hispanic Paradox when considering the context of history of health conditions and self-rated health. The Biosocial Theory incorporates the dynamic, bidirectional interactions between biological (e.g. health/illness) and social and contextual factors (nativity status). This theory also suggests that biological and social factors can be intersectional in nature and not work mutually exclusive. Within this study, the Biosocial Theory supports the notion that biology (e.g. medical conditions/diagnosis) social-cultural factors (nativity status) may play a role in this phenomenon. Additionally, contextual factors like self-rated health may also serve as a potential moderator around this relationship.

## Self-Rated health: a potential moderator

Self-rated health (SRH) is a reliable, valid, and dynamic indicator of health that is sensitive to health and social factors (e.g. engagement in social activities, physician-rated health status, alcohol use, and smoking behavior; Parkatti, Deeg, Bosscher, & Launer, 1998; Riosmena & Dennis, 2012) and has been primarily studied as a proxy measure for physical health outcomes in Latinx adults (National Center for Health Statistics. National Health Interview Survey, 2019; Lee, 2019). Research has shown that Mexican American immigrant status is significantly associated with poorer SRH compared to U.S.-born, non-Hispanic whites (National Center for Health Statistics. National Health Interview Survey, 2019; Tsai, Ford, Li, Zhao, & Balluz, 2010; Tejeda, Gallardo, Ferrans, & Rauscher, 2017). Interestingly, however, SRH’s accuracy in measuring Latinxs’ actual physical health has been contested by the literature. Studies have found that controlling for the language of the interview significantly reduces the differences in self-rated health between Latinxs and whites (Smith & Goldman, 2007; Bzostek, Goldman, & Pebley, 2007; Pettit & Gutierrez, 2018). Other scholars have suggested that a traditional cultural orientation among first-generation Latinxs may lead them to somatize psychological symptoms, contributing to findings that first-generation immigrants and Spanish speakers report lower SRH than their U.S.-born counterparts (Smith & Goldman, 2007; Pettit & Gutierrez, 2018).

For Latinx immigrants of Mexican origin, physical and mental health are often intertwined as researchers have found connections between the stressors prevalent within the Latinx immigrant population and physical and mental health outcomes. For example, perceived discrimination and minority stress predict both negative mental and physical health outcomes in Mexican-origin adults (Ai et al., 2017; Diaz-Santana et al., 2017; Ross et al., 2019). SRH is a reliable indicator of acculturative stressors in Mexican Americans and physical indicators of such stress (e.g. rises in cortisol levels and inflammation; Ai et al., 2017; National Center for Health Statistics. National Health Interview Survey, 2019), however, few studies have looked into its reliability in predicting poor mental health outcomes within this population. A study by Lommel, Thompson, Chen, and Waters (2019) found that poorer mental health was a significant predictor of worse SRH in Mexican Americans only, suggesting the presence of a biosocial factor influencing mental and physical health amongst Mexican American immigrants. Regardless, the association between self-rated physical health and mental health outcomes has been illustrated in studies on other populations, which indicates that SRH may be a useful proxy for common factors such as financial need, health burden, and health insurance status that impact overall health (Jones et al., 2018; Idler, Hudson, & Leventhal, 1999). Therefore, more studies are needed that examine whether SRH is a moderator for the Hispanic Paradox.

## History of health conditions: a potential mediator

Similarly, Latinx immigrants’ SRH conditions have been associated with factors known to predict poor mental health outcomes in Latinx adults. Common health conditions such as cancer, diabetes, and hypertension have been linked to immigrant stressors including but not limited to acculturative stress and processes, financial and status-related barriers to healthcare, and English language proficiency (Finch, Hummer, Kol, & Vega, 2001; Boen & Hummer, 2019; Moore, 2018). Researchers have also found associations between cancer, diabetes, and hypertension rates and mental health outcomes in immigrants. Analyses of the 2000 and 20001 National Health Interview Surveys (NHIS) have revealed that foreign-born immigrants reported fewer instances of chronic health conditions (e.g. hypertension, heart disease, asthma, cancer, and diabetes) than their U.S.-born counterparts, who do not seem to benefit from this health advantage (Hayes, 2017). More recent studies have suggested that mental health outcomes mirror this pattern: a study using data from the 2019 Canadian Chronic Disease Indicators (CCDI) and 2017 Canadian Community Health Survey found that immigrants reported higher mental health and lower chronic disease rates than non-immigrants, with the magnitude of this effect decreasing with their length of residency (Moon, Kim, Rote, Haley, & Sears, 2021).

A similar connection between nativity status and mental and physical health outcomes has been noted in Mexican immigrants. A 2008 study comparing both U.S.-born and immigrant individuals from four Hispanic-origin groups (Mexicans, Cubans, Puerto Ricans, and Dominicans) to White Americans found that Mexicans had better physical and mental health outcomes than all other groups regardless of nativity (Diaz, Koning, & Martinez-Donate, 2016). Moreover, U.S.-born Mexicans reported worse mental and physical health statuses than their Mexican-born counterparts, providing support for the Hispanic Paradox. Thus, chronic disease rates (namely cancer, diabetes, and hypertension) and mental health outcomes are intertwined, with nativity status offering a possible protective buffer against overall negative health outcomes in Mexican immigrants. Studies accounting for Mexican-origin immigrants’ nativity status in this physical and mental health outcome link are sparse, have arrived at mixed conclusions, and warrant further study (Spitzer, Kroenke, Williams, & Löwe, 2006; Kandula, Lauderdale, & Baker, 2007; Dey & Lucas, 2006).

## The present study

Researchers attempting to explain the relationships between nativity status, self-rated health, history of health conditions, and/or mental health outcomes often make use of prevalent theories such as Selection Theory, the Salmon Bias Hypothesis, and the Hispanic Paradox. However, the link between nativity status and mental health outcomes within Mexican immigrants remains largely understudied through the Biosocial Theory. In the present quantitative study, we primarily aim to replicate the associations between nativity status and mental health (i.e. depression and anxiety) among Mexican and Mexican American adults residing in the U.S. The secondary aim of this quantitative study is to examine if the history of health conditions mediated this relationship and self-rated health moderated the association. Utilizing national, cross-sectional data will allow us to bridge the literature surrounding physical and mental health amongst Mexican-origin adults in addition to providing a broader look at the pre-migration conditions contributing to the immigrant selection process.

## Methodology

### Participants

The participants for this study were abstracted from publicly released data files for the 2019 National Health Interview Survey (NHIS). The 2019 NHIS is a cross-sectional household interview survey that contains data from 33,138 households and aims toward being representative of Mexican-origin individuals due to its intentional oversampling of this population (Ma et al., 2014). The sampling method and protocol of this study have been described in greater detail elsewhere (Ma et al., 2014). For this investigation, we focused only on the sample of adult Mexican and Mexican American (*n* = 2,356) participants from the NHIS given the salience of the immigrant mental health paradox for this population. Table 1 presents descriptive statistics and intercorrelations for variables of interest in this study. The overall sample was 54.5% female, and the mean age was 42.64 years. Approximately 51.2% of the sample was born in the U.S. or a U.S. territory.

**Table 1.** Distribution of Study Variables for Mexican and Mexican American Participants

Variable	Overall	1	2	3	4	5	6	7
1. Born in U.S. (**%**)	51.20	–						
2. Female (**%**)	54.50	.03	–					
3. Age (**M, SD**)	42.64 (16.75)	-.21***	.05*	–				
4. Hx Health Conditions (**M, SD**)	.41 (.68)	-.02	.01	.50***	–			
5. Subjective Health (**M, SD**)	3.56 (1.11)	.04	-.06**	-.33***	-.43***	–		
6. Anxiety Symptoms (**M, SD**)	1.86 (3.58)	.16***	.11***	-.04*	.12***	-.26***	–	
7. Depressive Symptoms (**M, SD**)	2.21(3.81)	.14***	.08***	.02	.19***	-.34***	.79***	–

Note: *p < .05 **p < .01 ***p < =.001

### Measures

#### Nativity status

Nativity status was measured by one item: ‘Were you born in the U.S. or a U.S. territory?’ Responses included 1 = Yes; 2 = No; 7 = Refused; 8 = Not Ascertained; 9 = Don’t Know. Ninety-seven percent of the sample responded to this item. For 2.7% of the sample, this question was not ascertained and 0.3% of the sample refused or did not know. For analysis, we created a dichotomous variable where 1 = yes and 0 = no.

#### Mental health

The 2019 NHIS includes two mental health scales measuring symptoms of a generalized anxiety disorder (GAD) and depression. The set of questions in the GAD-7 and PHQ-8 ask adults to assess how often they have been bothered over the last 2 weeks by a set of specific symptoms. Response options to both GAD-7 and PHQ-8 questions are the same: (0) not at all, 1) several days, 2) more than half the days, and 3) nearly every day. These response categories correspond to 0–3 points, respectively, when scoring each question (Ma et al., 2014). For the present study, new variables were computed by summing the points for each question to produce a total score between 0 and 21 for the GAD-7 and between 0 and 24 for the PHQ-8 for each participant.

**Anxiety Symptoms.** Symptoms of generalized anxiety disorder (GAD) were measured using the 7-item Generalized Anxiety Disorder scale (GAD-7; Noyola, Moreno, & Cardemil, 2020). This is a brief scale to screen for GAD symptoms in the past two weeks and assess its severity in clinical settings and the general population (Viruell-Fuentes, Morenoff, Williams, & House, 2011; Moreno, Nelson, & Perrin, 2020). The GAD-7 has also been found to have moderately good operating characteristics for three other anxiety disorders – panic disorder, social anxiety disorder, and post-traumatic stress disorder (Cuevas, O’Brien, & Saha, 2017). The GAD-7 was developed based on the most correlated items with a 13-item scale that included 9 items from the criteria for GAD in the Diagnostic and Statistical Manual for Mental Disorders, Fourth Edition (DSM-IV) and 4 items based on a review of existing anxiety scales (Noyola et al., 2020). The internal reliability for this sample was very good (α = .90).

**Depressive Symptoms.** Symptoms of depression were measured using the 8-item Patient Health Questionnaire depression scale (PHQ-8; Leong, Park, & Kalibatseva, 2013). This is a valid diagnostic and severity measure for current depressive disorders derived using the nine-item criteria for depressive disorders in the DSM-IV. Symptoms of depression were assessed over the past two weeks. The PHQ-8 is used in clinical settings and population-based studies to screen for possible symptoms of clinically significant depression and to assess the severity of depressive symptoms (Leong et al., 2013). The PHQ-8 is an abbreviated version of the nine-item PHQ-9 scale (Nelson, Perez, Alcaraz, Talavera, & McCarthy, 2007) which excludes the question about thoughts of death and self-injury, an indicator of possible suicide risk. The internal reliability for this sample was good (α = .86).

#### History of health conditions

We investigated three self-reported health conditions (i.e. cancer, diabetes, and hypertension) related to the Hispanic Paradox for this analysis. For all health conditions, participants were asked, ‘Have you ever been told that you had diabetes, cancer, and hypertension. Responses included 1 = Yes; 2 = No; 7 = Refused; 8 = Not Ascertained; 9 = Don’t Know. We created dichotomous variables for each variable where 1 = Yes and 0 = No. We then computed a sum score to create a composite variable with a range of 0–3 for each participant.

#### Self-Rated health

Self-rated health was measured using one item, ‘How would you rate your general health?’. Responses included 1 = Excellent; 2 = Very Good; 3 = Good; 4 = Fair; 5 = Poor. We reverse-coded this item; higher scores indicate favorable self-rated health.

### Procedure

The NHIS is a cross-sectional household interview survey. The Sample Adult section of the 2019 NHIS covers a range of health topics including detailed information about the individual participant’s Health Status and Conditions (HSC), Functioning and Disability (FD), Pain and Pain Management (PPM), Health Care Access, and Health Service Utilization (HCAHSU), Health-Related Behaviors (HRB), Mental Health (MH), and Preventive Care (PC). In this study, only select data from the HCAHSU and MH sections were used along with select demographics. The target population for the NHIS is the civilian noninstitutionalized population residing within the 50 states and the District of Columbia at the time of the interview. Persons excluded from the NHIS are those with no fixed household address, active-duty military personnel and civilians living on military bases, persons in long-term care institutions, persons in correctional facilities, and U.S. nationals residing in foreign countries. The NHIS uses geographically clustered sampling techniques designed in such a way that each month’s sample is nationally representative, and data is collected continuously from January to December each year. Nationally, about 750 interviewers are trained and directed by health survey supervisors in the U.S. Census Bureau Regional Offices to conduct interviews for the NHIS.

The NHIS interview is conducted using computer-assisted personal interviewing (CAPI) that guides the interviewer through the questionnaire, automatically routing them to appropriate questions based on answers to previous questions. A ‘sample adult’ from each household with at least one household member aged 18 years or older is randomly selected by the computer to complete more detailed health-related questions about themselves. For this paper, ‘sample adult’ individuals will be referred to as study participants.

### Data analytic strategy

We examined normality, skewness, and kurtosis for our variables of interest. Symptoms of depression and anxiety, our dependent variables of interest, were not normally distributed in our sample. However, our main analyses were conducted utilizing the PROCESS Macro, which utilizes bias-corrected confidence interval estimations, robust standard errors, and 5,000 bootstrap estimates, which do not require normally distributed data (Huh, Prause, & Dooley, 2008; Hayes, 2017). Moreover, given the capacity of PROCESS to handle non-normally distributed variables, our large sample size, and ease of interpretation, we did not transform symptoms of anxiety or depression in subsequent analyses. Next, we conducted bivariate correlations to investigate the relationships between our variables of interest. Finally, we used the PROCESS (Model 5) Macro Version 3.4.1 (Huh et al., 2008), to conduct two moderated mediation models with nativity status on depression and anxiety through the history of health conditions with self-rated health as a moderator. Ninety-five (95%) confidence intervals from 5,000 resamples were generated by bias-corrected bootstrapping methods to determine the significance of moderated mediation. An indirect effect is considered significant if 0 does not fall between the calculated confidence intervals. All statistical analyses were conducted in SPSS Version 27.0; moderated mediation analyses, including syntax and data for probing and visualizing interactions, were generated in PROCESS Macro Version 3.4.1 in SPSS Version 27.0 (Huh et al., 2008; Hayes, 2017). For the present paper’s STROBE (Strengthening the Reporting of OBservational studies in Epidemiology) guidelines checklist, please refer to Table S1.

## Results

### Preliminary analyses

We present means, standard deviations, and correlations among variables of interest in Table 1. Several one-way analyses of variance (ANOVA) revealed significant differences in anxiety and depression by sex with females reporting higher symptoms of anxiety and depression compared to males (data not shown). Bivariate Pearson correlations revealed significant positive associations between nativity status and both symptoms of anxiety (*r* = .16, *p *< .001) and depression (*r* = .14, *p *< .001). Nativity status was also inversely associated with age (*r* = -.21, *p *< .001). Sex (i.e. female) was negatively associated with self-rated health (*r* = -.06, *p = *.007). and positively associated with age (*r* = .05, *p *= .03) as well as symptoms of anxiety (*r* = .11, *p *< .001) and depression (*r* = .08, *p *< .001). Age was positively associated with history of health conditions (*r* = .50, *p *< .001) and negatively associated with self-rated health (*r* = -.33, *p *< .001) and symptoms of anxiety only (*r* = -.04, *p = *.04). History of health conditions was negatively associated with self-rated health (*r* = -.43, *p *< .001) and positively associated with symptoms of both anxiety (*r* = .12, *p *< .001) and depression (*r* = .19, *p *< .001). Furthermore, self-rated health was inversely associated with symptoms of anxiety (*r* = -.26, *p *< .001) and depression (*r* = -.34, *p *< .001). Given the significant associations between sex and symptoms of anxiety and depression as well as the significant correlation of age with variables of interest, we controlled for these variables in all subsequent analyses.

### Main analyses

**Figure 1.**
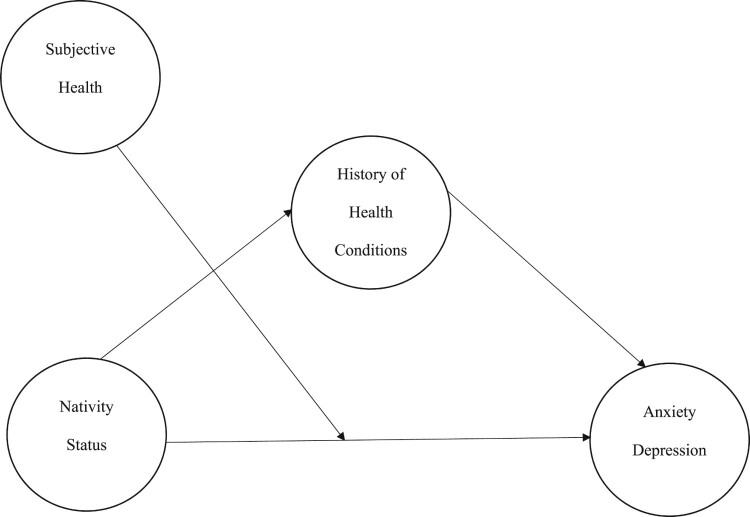
Conceptual Model for Moderated Mediation

**Figure 2.**
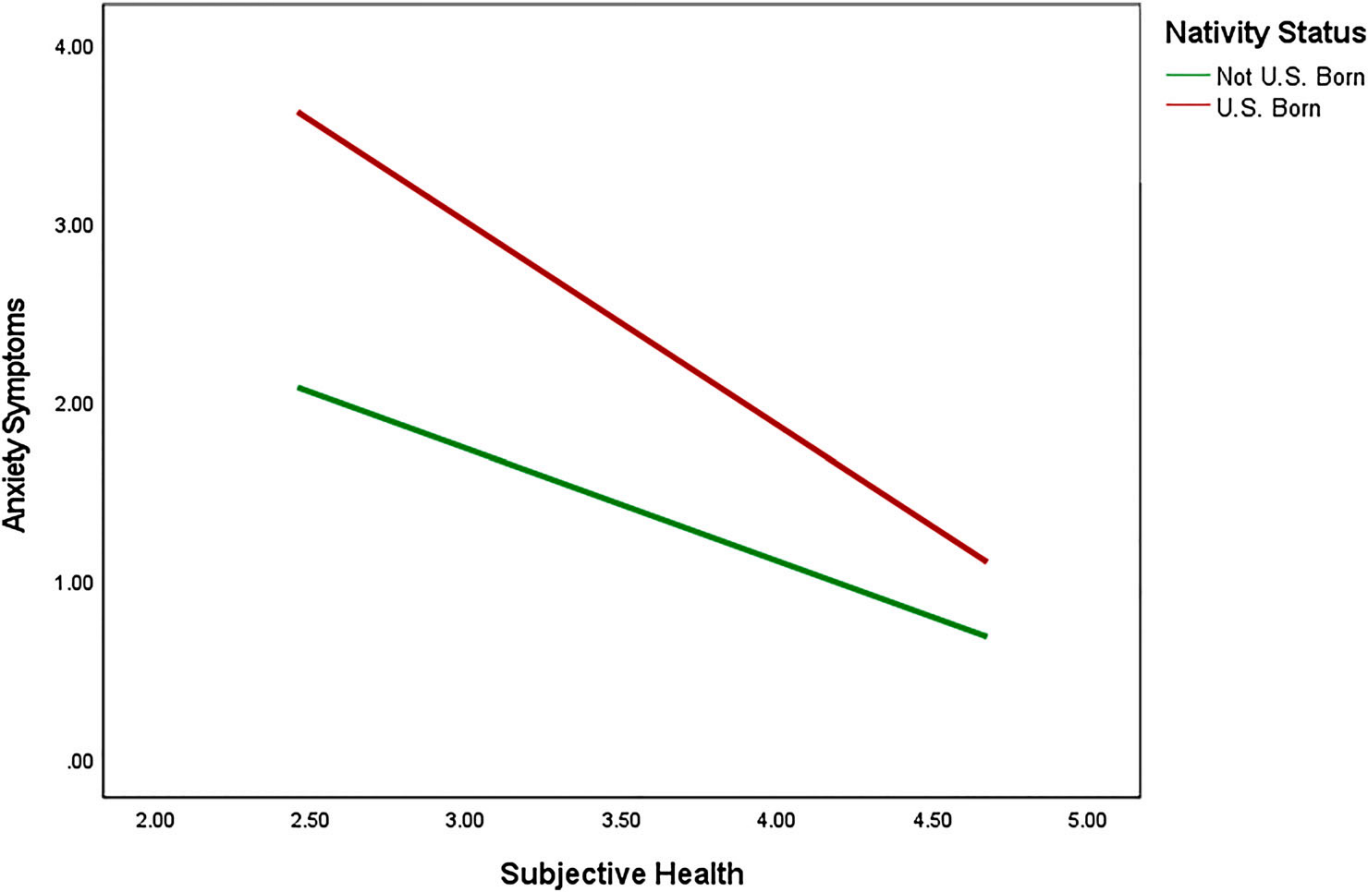
The Interaction Between Nativity Status and Subjective Health on Anxiety Symptoms

**Figure 3.**
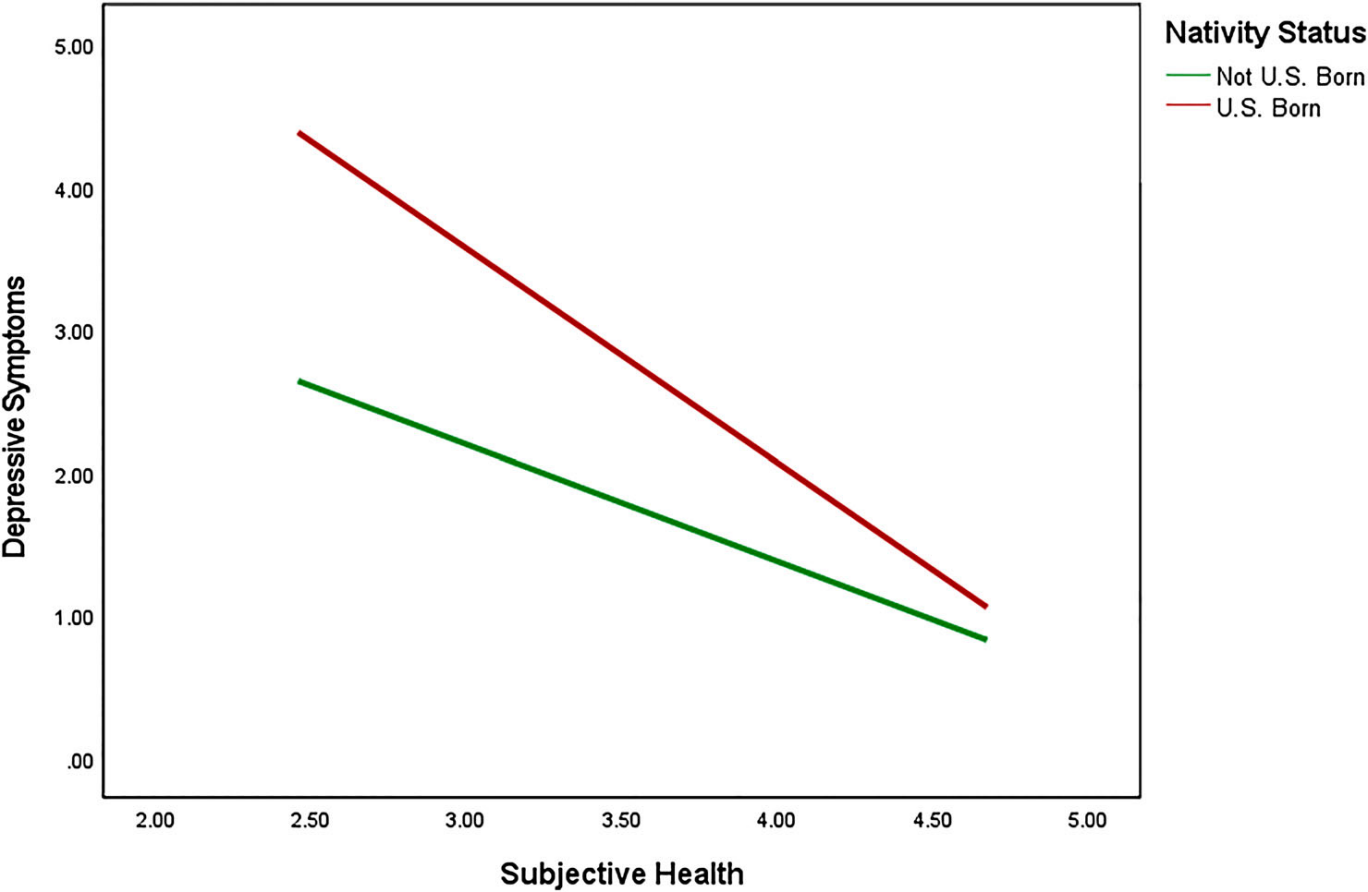
The Interaction Between Nativity Status and Subjective Health on Depressive Symptoms

We used PROCESS (Huh et al., 2008; Model 5) to examine two moderation mediation models of nativity status on anxiety and depressive symptoms through the history of health conditions with self-rated health as a moderator (see Figure 1 for conceptual model). Results of these moderated mediation analyses are shown in Tables 2 and 3. Consistent with our hypotheses, results indicated that the association between nativity status and anxiety was explained (i.e. mediated) by the history of health conditions (*B* = .05, 95% CI: .01, 09). Additionally, the association between nativity status and depression was mediated by the history of health conditions (*B* = .06, 95% CI: .02, .12). With regard to moderation, results indicated that the cross-product term between nativity status and self-rated health was significant for both anxiety (*B* = -.51, *t* = −3.96, *p* < .001) and depression (*B* = -.68, *t* = −5.14, *p *< .001), indicating that self-rated health was a significant moderator in both mediation models. To understand these findings, we probed the conditional effects of nativity status on anxiety and depression at values of the moderator, self-rated health. Findings indicated significant differences in anxiety symptoms by nativity status at varying levels of self-rated health (see Figure 2). Moreover, participants who were not born in the U.S. had overall lower levels of anxiety at all levels of self-rated health. Similarly, findings revealed significant differences in depressive symptoms by nativity status at varying levels of self-rated health (see Figure 3). In general, participants who were born in the U.S. had lower levels of depressive symptoms at varying levels of self-rated health except for when self-rated health was high. In other words, when self-rated health was high, there was no difference in depressive symptoms among participants who were born in the U.S. and born outside of the U.S.

**Table 2.** Mediation Model of Nativity Status on Anxiety Symptoms Through History of Health Conditions with Subjective Health as a Moderator

	*B*	*SE*	*t*	*p*	95% Confidence Interval
	Hx Health Conditions				
Nativity Status	.11	.02	4.56	< .001	(.06, .16)
	Anxiety Symptoms				
Nativity Status	2.79	.48	5.82	< .001	(1.85, 3.73)
History of Health Conditions	.42	.13	3.31	.001	(.17, .67)
Subjective Health	-.63	.09	−6.57	< .001	(-.82, -.44)
Nativity Status * Subjective Health	-.51	.13	−3.96	< .001	(-.76, -.26)
Indirect Effect of Nativity Status	.05	.02	–	–	(.01, .09)

Note: Controlled for age and sex

**Table 3.** Mediation Model of Nativity Status on Depressive Symptoms Through History of Health Conditions with Subjective Health as a Moderator

	Hx Health Conditions				
	*B*	*SE*	*t*	*p*	95% Confidence Interval
Nativity Status	.12	.02	4.74	< .001	(-.62, -.45)
	Depressive Symptoms				
Nativity Status	3.42	.50	6.88	< .001	(2.45, 4.40)
History of Health Conditions	.55	.13	4.14	< .001	(.29, .81)
Subjective Health	-.82	.10	−8.26	< .001	(−1.01, -.62)
Nativity Status * Subjective Health	-.68	.13	−5.14	< .001	(-.94, -.42)
Indirect Effect of Nativity Status	.06	.02	–	–	(.02, .12)

Note: Controlled for age and sex

## Discussion

Given the consistent literature on the Hispanic Paradox, the purpose of this study was to replicate the associations between nativity status on depression and anxiety. We then examined if the history of health conditions mediated this relationship and if self-rated health moderated the association. Results found a significant direct association between nativity status and anxiety and depression. These results support the overall literature as the Hispanic Paradox has consistently shown to have similar effects for anxiety and depression (e.g. Cooper, Bachem, Meentken, Aceves, & Perez Barrios, 2020a). The fact that our study was consistent with the overarching literature on anxiety and depression is interesting yet suggests that nativity status is directly associated with symptoms of anxiety-related symptomatology (e.g. constant worry, uncontrollable thoughts, heart palpitation) and mood-related symptoms (e.g. sadness, helplessness, hopelessness, worthlessness, etc.). Additionally, the association between nativity status and depression was mediated by the history of health conditions. This finding is conceptually sound given that the Hispanic Paradox of depression is prevalent by means of history of health conditions. Additionally, our study sheds light on associations and suggests how this paradox may not be associated with all types of mental health disorders.

Concerning moderation, results demonstrated a significant interaction between nativity status and self-rated health for both anxiety and depression, indicating that self-rated health was a significant moderator in both models. These findings evidenced significant differences in anxiety symptoms by nativity status at varying levels of self-rated health. For example, participants who were born outside the U.S. had overall lower levels of anxiety at all levels of self-rated health. Similar to anxiety outcomes, findings revealed significant differences in depressive symptoms by nativity status at varying levels of self-rated health. However, the direction of the associations countered what was expected. Our findings revealed that participants who were born in the U.S. had higher levels of depressive symptoms at varying levels of self-rated health except for when general health was high. In other words, when self-rated health was high, there was no difference in depressive symptoms among participants who were born in the U.S. and born outside of the U.S. (See Table 3). Several psychological theories may explain this phenomenon. For example, higher self-efficacy and an internal locus of control have been linked to more positive perceptions of self-rated health (Min, Rhee, Lee, Rhee, & Tran, 2014; Min et al., 2014; Shor, Roelfs, & Vang, 2017) and adherence to overall health promotion (Shor et al., 2017). Therefore, when high self-rated health is reported to the Hispanic Paradox for depression, there is no significant difference between both nativity statuses.

### Clinical implications & Future directions

The reported findings may have significant implications not only for mental health professionals but also for general care providers who work with Latinx communities. Culturally competent services are key to the overall health and well-being of Latinx individuals in the U.S. (Luszczynska, Schwarzer, Lippke, & Mazurkiewicz, 2011). In particular, understanding how the Hispanic Paradox may play a role in this community’s care. Providers must consider how the paradox may bias perceptions of health. The findings in this study are consistent with the literature among Mexican-born and U.S.-born Mexican-Americans and the paradox, however, health and well-being have many within-group differences among other Latinx subgroups (Commodore-Mensah et al., 2018; Kroenke, Spitzer, & Williams, 2001; Kroenke, Spitzer, Williams, & Löwe, 2010). Therefore, providers should not overgeneralize the health status of their Latinx identifying patients, specifically for foreign-born Latinxs, and assume that they have lower rates of anxiety, depression, and better physical health. The Latinx community battles with many logistical barriers to care (e.g. transportation, lack of Spanish-speaking providers; Kroenke et al., 2010), thus, it is important that providers remain culturally competent to ensure they assist in minimizing logistical and cultural barriers by checking their biases before working with the community. As it can be difficult for providers to address structural barriers to care (e.g. lack of health insurance) on an individual level, it is vital they meet Latinx clients where they are.

It should be noted that, more recently, the Hispanic paradox literature has expanded on theories beyond generation status, the Salmon Bias, or Selective Theory that impact whether or not immigrants experience negative physical or mental health outcomes, particularly analyzing the role of resilience (Kroenke, Spitzer, Williams, Monahan, & Löwe, 2007; Gonzalez-Guarda, Stafford, Nagy, Befus, & Conklin, 2020; Kroenke et al., 2009). It is necessary to highlight that the Latinx community is resilient and upholds many cultural values as strengths, rather than deficits (Stronks et al., 2020; Foo et al., 2018). Providers should emphasize these protective factors by adapting interventions to include relevant cultural values supported in the research literature, such as integration of family into care (e.g. Woodward et al., 2012), utilization of religiosity (e.g. Lommel et al., 2019; Villarreal et al., 2017), and increasing social support (e.g. Flores et al., 2008).

Future research can expand upon the association between history of health conditions and specific mental health disorders by highlighting the specific types of physical and mental health conditions that may support the Hispanic Paradox. Given that our results suggest U.S-born Mexican individuals have higher rates of anxiety and depression symptomatology, future studies should consider analyzing not only nativity status but also nativity factors (e.g. region of origin, language, SES, etc.), attitudes (e.g. stigma, discrimination, acculturation, etc.), barriers such as environmental constraints (e.g. transportation, childcare, healthcare, etc.), lack of culturally competent services, and other socioeconomic and sociocultural factors that may uniquely impact Mexican American individuals’ mental health outcomes (National Center for Health Statistics. National Health Interview Survey, 2019; Sanchez, Vargas, Juarez, Gomez-Aguinaga, & Pedraza, 2017; Cooper et al., 2020).

Furthermore, to honor the resiliency of immigrant communities, research should be conducted in a strengths-based perspective to understand how protective factors may also play a moderating role in this association. Efforts to acknowledge and integrate the strengths of the Latinx community, rather than assume a deficit model, will aid in uplifting, better serving, and advocating for Latinx individuals. Future studies can also examine how self-efficacy and internal locus of control are associated directly with the Hispanic Paradox among Mexican-born and U.S.-born Mexican Americans. Lastly, due to the vast diversity among the Latinx and migrant populations, it is important to understand intergroup differences and explore within-group analyses across more contextual factors (e.g. other nationalities, gender identity, education, health literacy, etc.).

### Strengths and limitations

This study has several strengths and limitations. First, the use of data drawn from the 2019 National Health Interview Survey (Ma et al., 2014) provided a large, nationally representative sample of noninstitutionalized adults. However, the sample included in this study was homogenous in that participants all self-identified as Mexican. Therefore, the present study is cognizant of the differences between various Latinx subgroups (e.g. the substantial differences between Latinx subgroups in reporting SRH National Center for Health Statistics. National Health Interview Survey, 2019), and results herein do not generalize to all Latinxs. It is also critical to acknowledge the wide variation among Mexican and Mexican populations (e.g. racial identities such as ‘blanco’ or ‘mestizo’, native language, generational status, education level, legal status, etc.) that is likely to affect health and health outcomes within this population (Sanchez et al., 2017; Cooper et al., 2020a). Although this depth of analysis was out of this particular study’s scope, future studies may greatly benefit from analyzing these within-group differences in Mexican immigrants. It is also important to continue to understand the ways in which migrants and non-migrants worldwide appear to be susceptible to developing negative mental health outcomes (e.g. depression), which has been more recently studied (Alegría et al., 2008; Moreno & Cardemil, 2018), suggesting that this Hispanic Health Paradox extends further to the Immigrant Health Paradox (Garcia, Wilborn, & Mangold, 2017).

It should also be noted that the sample lacks representation of incarcerated adults, who are disproportionately members of minoritized groups (i.e. Black and Latinx adults; Moreno et al., 2021). The NHIS also provides critical data on other immigrant groups (e.g. African, Middle Eastern) that were not incorporated into this study, thus future research should highlight these communities as they are not well characterized in health disparities research (Barragán et al., 2020).

Methodologically, the cross-sectional design of the NHIS does not allow for causal inferences. Study variables were measured at the individual level, and structural factors such as the availability of health care services or area-level SES were not included in this study. Future research should investigate these structural barriers to better understand the social determinants of health faced by immigrant groups residing in the U.S. Since the NHIS relies on interviews, recall bias is likely to play a factor in either over- or under-reporting certain outcomes. Despite these limitations, the findings in this study are important to understanding how the Hispanic Paradox applies to Mexican and Mexican Americans in the U.S. amidst relevant covariates.

## Conclusion

Given the consistent literature on the Hispanic Paradox, the present study aimed to replicate the associations between nativity status on depression and anxiety among Mexican and Mexican American adults residing in the U.S. This paper is among the first to examine the impact of preexisting health conditions and self-rated health on the relationship between nativity status and mental health. Our study found support for the Hispanic Mental Health Paradox, where participants who were not born in the U.S. had lower levels of anxiety at all levels of self-rated health. This pattern held for depression at all levels of self-rated health, except for when self-rated health was high. Furthermore, Mexican and Mexican American individuals’ history of health conditions (particularly cancer, diabetes, and hypertension) may explain the relationship between nativity status and mental health outcomes.

Understanding the role that the Hispanic Paradox may play in the Latinx community's healthcare use and access is vital to closing the health disparity gaps that persist among this population. Future research may benefit from highlighting the specific types of physical and mental health conditions associated with the Hispanic Paradox. Future studies may also greatly benefit from analyzing the variation among Mexican and Mexican populations in order to better understand the many socio-cultural factors (e.g. racial identities such as ‘blanco’ or ‘mestizo’, native language, education levels, legal status, etc.) associated with the Hispanic Paradox. Regardless, these findings continue to shed light on our understanding of the Hispanic Paradox.

## Note

1 ‘Hispanic’ and ‘Latina/o’ are widely used interchangeably to refer to Latin Americans and/or Spanish-speakers. Due to growing awareness that ‘Latinx’ is additionally inclusive of the gender-expansive and Afro-Latinx communities, the author will use ‘Latinx’ in lieu of ‘Hispanic’ and ‘Latina/o’ to describe the sample/population, yet still refer to the Hispanic Paradox as its original named term. See Cardemil et al. (2019) for more information.
